# Transcriptome Sequencing Reveals the Genome Sequence of *Pea Early Browning Virus* from a 29-Year-Old Faba Bean Sample

**DOI:** 10.1128/MRA.00673-20

**Published:** 2020-07-09

**Authors:** S. Maina, L. Zheng, S. King, V. L. McQueen, S. L. Norton, B. Rodoni

**Affiliations:** a Australian Grains Genebank, Agriculture Victoria, Horsham, Victoria, Australia; b Agriculture Victoria, AgriBio, Melbourne, Victoria, Australia; c School of Applied Systems Biology, La Trobe University, Bundoora, Victoria, Australia; DOE Joint Genome Institute

## Abstract

*Pea early browning virus* (PEBV) is transmitted by soil-inhabiting trichodorid nematodes and via seeds. The transcriptome sequencing method, followed by *de novo* assembly, revealed the PEBV Libyan isolate LyV66-91 genome. Its RNA1 resembled that of UK isolate SP5 with 93.91% nucleotide identity, and its RNA2 had 63.32% nucleotide identity to that of Dutch isolate E116.

## ANNOUNCEMENT

*Pea early browning virus* (PEBV) (genus *Tobravirus*, family *Virgaviridae*) infects about 30 legume species in a nonsystemic manner (1) and causes systemic infections in field pea (Pisum sativum L.) and faba bean (Vicia faba L). Its genome consists of two positive-strand RNA molecules (RNA1 and RNA2), each encapsidated separately in a rod-shaped particle (2). The RNA1 molecules of all three species within the *Tobravirus* genus are similar in length, but the lengths of their RNA2 molecules differ considerably (3–5). PEBV is transmitted mainly by five soilborne nematodes within the family *Trichodoridae* (6, 7), and its strains have been found in Europe, North Africa, and the Horn of Africa (7). PEBVs consist of three strains, Dutch, British, and probably Italian No. 6 (1, 8, 9). PEBV is highly seedborne in field pea and Vicia faba L. (broad bean or tick bean) (10). Seed transmission of PEBV was reported in 1962 in the pea cultivar Rondo and in faba beans (11).

Plants infected by PEBV have diverse host reactions; for instance, peas can be asymptomatic or can exhibit foliar symptoms ranging from brown or necrotic spots to plant death (11, 12). However, infected faba bean plants have been found to be mostly asymptomatic (13). PEBV is listed as a quarantinable pathogen in the Australian Government Biosecurity Import Conditions. Therefore, imported pulse seeds are required to be thoroughly assessed through detailed visual assessment and molecular testing before seed lines can be released in Australia. Despite the potential threat of PEBV to the Australian pulse industry, there is currently only one PEBV genome sequence available in GenBank (6).

Isolate LyV66-91 was obtained from a faba bean crop in Libya in 1991 (14) and has been maintained at −20°C in the Post-Entry Quarantine (PEQ) facility in Horsham, Australia. Its RNA was extracted using a ZR plant RNA miniprep kit, and a quality control check was done, followed by library preparation using a TruSeq stranded total RNA sample preparation kit with Ribo-Zero Plant (Illumina, San Diego, CA), as described previously (15). The library was diluted, denatured, and then sequenced using a MiSeq v3 kit (Illumina) with paired-end reads (2 by 251 cycles).

Quality control of the raw read fastq files was performed using Trim Galore (16) with the minimum sequence length set to 50 bp and the minimum required adapter overlap (stringency) set to 1 bp. The *de novo* assembly was performed using the metaSPAdes v3.13.0 genome assembler (17) with default settings. In addition, a second assembler, CLC Genomics Workbench 20 (CLC bio, Qiagen), was used as described previously (15), with the minimum contig length set to 800 bp. The contigs of interest were imported into Geneious (18), multiple alignment and percent nucleotide identity with GenBank sequences were derived using MUSCLE (19), and annotation was performed with transfer annotation selected and similarity set at 90%, while other settings were left as defaults (18).

The sequencing run yielded 6,263,484 raw reads, and 6,080,125 reads remained after quality control. The CLC Genomics Workbench *de novo* assembly generated 244 contigs. All the CLC Genomics Workbench-derived contigs were subjected to a homology BLASTn search using BLAST+ v2.7 (20), which revealed two contigs resembling RNA1 and RNA2 of PEBV. The contig matching PEBV RNA1 consisted of 7,037 nucleotides with 235,443 reads mapping to it with an average coverage depth of 3,899× and a GC content of 40.7%. The RNA2 contig consisted of 2,604 nucleotides with 1,252,888 reads mapping to it with an average coverage depth of 5,606× and a GC content of 42%. Isolate LyV66-91 RNA1 had 93.91% nucleotide identity to UK PEBV isolate SP5 (GenBank accession number X14006), currently known as the British strain. Although biological studies conducted by Bos et al. (14) determined that the host range and symptoms caused by isolate LyV66-91 are very similar to those of the Dutch type strain isolate E116 (GenBank accession number AJ006500), the two RNA2 genomes shared 63.32% nucleotide identity. Because the RNA1 genome sequence of the Dutch type strain E116 is not available in GenBank, we were unable to compare the two isolates at genomic level.

This study has significant value to the Australian pulse biosecurity industry in relation to exotic virus entry into Australia. The current genome sequences will lead to enriching current diagnostic markers for PEBV and enhance its molecular testing within the Horsham PEQ facility and elsewhere. In addition, the PEBV genomic data presented in this study will inform future studies and stimulate new research to understand recombination and genetic connectivity among PEBV strains, potential adaptations to enable infection of leguminous hosts, and their vector spectra in relation to their phylogenetic groupings.

### Data availability.

The final sequences were deposited in DDBJ under accession numbers LC528622 and LC528623. The raw reads were deposited in the NCBI SRA under accession numbers SRX7814135 and SRX7814136 and BioProject number PRJNA607790.
